# Impact of Incremental Antiseptic Selection on Antibiotic Cross‐Resistance and Enhanced Biofilm Formation in *Escherichia coli* and *Acinetobacter baumannii*: In Vitro

**DOI:** 10.1155/ijm/5044102

**Published:** 2026-09-26

**Authors:** Megha Prasad, Pratheesh N. Prabhakaran, Kulpreet Bhui, Deepa Murali

**Affiliations:** ^1^ Savlon Swasth India Mission, ITC Life Sciences and Technology Centre, Bangalore, India

## Abstract

Chlorhexidine, cetrimide, chloroxylenol, and povidone iodine, in various combinations and concentrations are widely used antiseptics in clinical settings for skin antisepsis and hand hygiene in India. Evidences suggest that exposure of bacterial pathogens to antiseptics over a period of time can result in adaptation of bacteria resulting in tolerance and cross‐resistance towards antiseptics and antibiotics. Given the crucial role that antiseptics play in reducing infection and preventing the spread of AMR, understanding the adaptation to antiseptics becomes a critical element in using them effectively. This study is aimed at determining the behavior of two clinically relevant species *Escherichia coli* and *Acinetobacter baumannii* adapted to chlorhexidine (CHG), cetrimide, chloroxylenol (PCMX), and povidone iodine (PVP‐I). In particular, we have evaluated changes in minimum inhibitory concentration (MIC), cross‐tolerance/resistance to both antibiotics and antiseptics and biofilm formation capability post the evolution study towards respective biocides. Both the studied bacterial species adapted to concentrations of antiseptics higher than their initial MICs. Among the adapted strains, *E. coli* cetrimide showed tolerance to cetrimide with a 16‐fold increase in MIC. Similarly, a 4‐fold increase in MIC against PVP‐I was observed in *A. baumannii* PVP‐I strain, indicating tolerance to PVP‐I. Cross‐adaptation studies indicated cross‐tolerance of *E. coli* CHG and *E. coli* cetrimide to cetrimide and CHG, respectively. Among seven antibiotics tested, *A. baumannii* PCMX showed antibiotic resistance towards tetracycline and imipenem. A concomitant change in biofilm formation, indicated by an increased specific biofilm formation value was also observed. Moreover, CHG, cetrimide and PVP‐I adapted *A. baumannii* strains showed sensitivity towards antibiotics, gentamicin, piperacillin, and tetracycline, a functional trade‐off for survival at high antiseptic concentrations. Collectively, these in vitro findings suggest that the decision to use appropriate antiseptics should be based not solely on antiseptic and antibiotic susceptibility data, but also on pathogens to be neutralized, and specific phenotypic factors like bacterial fitness and biofilm‐formation capabilities. Thus, frequent clinical microbiological monitoring with respect to antiseptic usage is crucial in controlling infection and spread of AMR.

## 1. Introduction

Disinfectants and antiseptics have become an integral part of infection control practices and play an important role in the prevention of nosocomial infections [1] especially those caused by ESKAPE pathogens (*Enterococcus faecium*, *Staphylococcus aureus*, *Klebsiella pneumoniae*, *Acinetobacter baumannii*, *Pseudomonas aeruginosa*, and *Enterobacter* spp.) [2] and *Escherichia coli* [3, 4]. Antiseptics are extensively used in healthcare, targeted home/personal hygiene, community environments, such as day care and senior citizen facilities, and industrial facilities for processing of food and water [5].

They are known to have a broader spectrum than antibiotics and interact with multiple targets in the bacterial cells resulting in irreversible bactericidal or reversible bacteriostatic effects [1, 6]. They primarily target the cytoplasmic membrane and bacterial enzymes [6]. Some of the most commonly and extensively used antiseptics are chlorhexidine gluconate (CHG), povidone‐iodine (PVP‐I) [7], and chloroxylenol (PCMX) [8]. Between them, they represent different mechanisms of action, cationic antiseptics, oxidizing agents, and phenolics, respectively. Briefly, CHG being a positively charged hydrophobic and lipophilic molecule interacts with the cell membrane of bacteria, mainly with the phospholipids and lipopolysaccharides. This allows for the CHG to penetrate the cell, altering the cell′s osmotic equilibrium, and damaging and compromising the cellular integrity [9]. PVP‐I, an iodophor consisting of a complex iodine and polyvinylpyrrolidone exists as active free‐iodine and the PVP‐I complex in aqueous solution. Iodine can penetrate rapidly into microorganisms and oxidize nucleotides, proteins, and fatty acids, leading to cell death [10]. PCMX is a phenolic antiseptic that shows similarity to other phenolic antibacterial agents like triclosan and hydroquinone in its mechanism of action. They are assumed to function by membrane disruption causing cell leakage. They could also act on the cytoplasmic membrane causing protein denaturation [11, 12].

Studies have shown that repeated exposure of specific gram‐negative, nosocomial infection‐causing bacteria towards antiseptics results in emergence of antiseptic tolerance with increased antiseptic MICs [13]. For instance, study by Sultan and Ahmed [14] showed that of the total studied clinical S*taphylococcus aureus* isolates, 25.9% showed a decreased susceptibility to chlorhexidine. Among the chlorhexidine tolerant isolates, methicillin‐resistant *Staphylococcus aureus* (MRSA) strains had a higher MIC, 0.5–16 mg/mL over methicillin‐susceptible *Staphylococcus aureus* (MSSA) strains, 0.5–8 mg/mL.

Apart from antiseptic tolerance, cross‐resistance towards antibiotics is a bigger threat. For example, the study by Guo et al. [15] observed that exposure to environmental concentrations of 0.20–1 mg/mL of PCMX accelerates the intergeneric conjugative transfer of antibiotic resistance genes by 9‐fold in *E. coli*. Similarly, it has been observed that clinical strains of *Klebsiella pneumoniae* that acquire CHG tolerance exhibit cross‐resistance towards colistin [16].

Although, studies comparing the susceptibility patterns of clinical*E. coli* and *Acinetobacter baumannii* strains towards CHG and PVP‐I have been previously reported [13, 17], correlational studies exploring the cross‐resistance of *E. coli* and *A. baumannii* adapted to CHG, cetrimide, PCMX, and PVP‐I towards each other, and antibiotics has previously not been evaluated. In this study, we are primarily aimed at determining the cross‐resistance profile of *E*. *coli* ATCC 10536 and *Acinetobacter baumannii* ATCC 19606 on extended exposure to PCMX, PVP‐I, CHG, and cetrimide. Moreover, we have also quantified the effect of cross‐adaptation/tolerance on the biofilm forming potential of these adapted strains.

## 2. Material and Methods

### 2.1. Bacterial Species and Growth Media

*E. coli* ATCC 10536 and *A. baumannii* ATCC 19606 were subcultured in tryptic soy broth (M011‐500G, HiMedia) and maintained in tryptic soy agar (M1968, HiMedia) plates. The samples for antibiotic susceptibility study were cultured in Mueller–Hinton broth (MH) (M391‐500G) and Mueller–Hinton agar (MHA) (M173‐500G).

### 2.2. Antiseptic Stock Solutions

CHG (RN Laboratories Ltd., India), cetrimide (Kenoor Organics Ltd., India), chloroxylenol (UniLab, India), and povidone iodine (Ashland Global, United States) were the actives studied. A 100 mg/mL stock solution of CHG, cetrimide and PVP‐I, respectively were made in sterile dH_2_O for adaptation studies. For PCMX, a stock solution of 1 mg/mL concentration was prepared by initially dissolving the active in 1 mL of absolute ethanol followed by resuspension in 9 mL of MH broth and 30 mL of dH_2_O. The stock concentrations of CHG, cetrimide, and PVPI used for MIC studies were 15, 30, and 100 mg/mL, respectively.

### 2.3. Antibiotic Susceptibility and MIC Determination

The antibiotic susceptibility and MIC testing for the bacterial species were assessed using the Ezy MIC strip (HiMedia). The MIC breakpoints were determined as per the interpretative criteria provided by CLSI M100 [18, 19]. The antibiotics used were gentamicin (EM061I‐30ST), meropenem (EM080I‐30ST), ciprofloxacin (EM082I‐30ST), tetracycline (EM056I‐30ST), ceftazidime (EM012I‐30ST), piperacillin (EM041I‐30ST), imipenem (EM104I‐30ST), cefotaxime (EM064I‐30ST), colistin (EM020I‐30ST), and cefepime (EM070I‐30ST). Briefly, the bacterial density of *E. coli* and *A. baumannii* was adjusted to the McFarland 0.5 turbidity standard. A sterile cotton swab was used to swab the MHA plates. The readymade strips containing the gradient concentration of the respective antibiotics were applied. The plates were incubated at 37°C overnight, and the MIC value was noted and interpreted using the CLSI chart. The antibiotic susceptibility and MIC breakpoint estimation were performed pre‐ and postadaptation to the active agents.

### 2.4. Antiseptic MIC Determination

The minimum inhibitory concentration required to inhibit the growth of the tested microorganisms was estimated using a modified 96‐well based microdilution method [18, 20]. The antimicrobial agents were serially diluted two‐fold in sterile 96‐microwell plates (Cat No. 3596, Corning) except in the wells demarcated for bacterial growth control. The starting concentrations for the antiseptic agents used in the study are as follows: CHG 7.5 mg/mL, cetrimide 15 mg/mL, PVP‐I 50 mg/mL, and PCMX 500 *μ*g/mL. A total of 100 *μ*L of the microbial culture with ~10^6^ CFU/mL microbial load was added to each well except in negative control wells. The plates were incubated at 37°C for 16–18 h. After incubation, 0.02% of resazurin was added to each well and incubated for 30–60 min. Color changes were recorded with a fluorescence reading at 530–590 nm using Tecan Infinite M Plex multimode reader (Bioscreen, Tecan Trading AG, Seestrasse, Männedorf, Switzerland).

### 2.5. Adaptation of *E. coli* and *A. baumannii*

**Figure 1:**
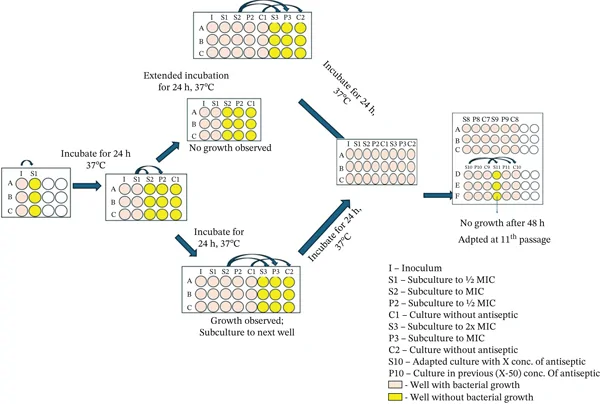
Flowchart depicting the method used for adaptation of bacterial strains to antiseptic agents.

*E. coli* and *A. baumannii* samples were serially subcultured to increasing concentrations of the antiseptics in a 48‐well plate (Cat No. 3548, Corning). For CHG and cetrimide adaptation, each 1‐mL volume consisted of 980 *μ*L of TSB, 10 *μ*L of antiseptic, and 10‐*μ*L bacterial stock. For PCMX and PVP‐I, the volume of antiseptic and bacterial stock changed according to the concentration of the antiseptic used for adaptation. The antiseptic concentrations used initially for each bacterium were selected to be below their respective MICs (1/2 MIC values). The concentration of each respective antiseptic was initially increased by factor of 2 and followed by an increase of 5, 10, 50, 100, or 1000 *μ*g/mL (Table S1) depending on the progression of the adaptation. All treated samples were incubated initially for 24 h at 37°C and assessed for growth. In case of no growth or low growth after 24 h, the samples were incubated for additional 24 h at 37°C and again assessed visually for growth. For each of the passages, three concentrations of antiseptics were inoculated in parallel as follows: the same concentration as previous passage, incremental concentration, and no antiseptic. Post adaptation, the bacterial samples were subcultured at the adapted concentration for 1 week, followed by 5 passages in antiseptic‐free TSB to stabilize the adaptation. The bacteria were then grown back in media supplemented with antiseptic, at respective adapted MICs for each strain, to check for stability of the adapted strains. Adaptation to each antiseptic was conducted on the same three biologically independent replicates (Figure 1).

### 2.6. Biofilm Formation

Bacterial strains were studied for their ability to form biofilms using quantitative biofilm assay as described by Gomez et al. [21] with modifications. On a sterile 96‐well plate (Cat No. 3596, Corning), the actives CHG, cetrimide, PCMX, and PVP‐I were serially diluted in triplicates. The bacterial culture of OD_600nm_ 0.2 ± 0.05 was diluted in the ratio 1:100 and then added to the wells with the actives. The wells, G1 to G12 were kept as positive control (bacteria only) and the wells, H1–H12 were kept as negative control (media only). The plate was incubated for 48 h at 37°C for biofilm formation. Following incubation, the plates were washed 3x with PBS, after which 0.2% of crystal violet dye was added and incubated for 15 min. The plate was then washed thrice with sterile distilled water to remove excess stain. The adhered dye was resolubilized using 30% acetic acid. The absorbance in each well was measured at 600 nm using the Tecan Infinite M Plex multimode reader (Bioscreen, Tecan Trading AG, Seestrasse, Männedorf, Switzerland). The negative control′s OD_600nm_ value was kept as the OD cut‐off (ODC) value. Based on the OD_600nm_, the strains were classified as weak biofilm producers if ODC < OD ≤ 2 × ODC, moderate biofilm producers if 2 × ODC < OD ≤ 4 × ODC, and strong biofilm producer if OD > ODC × 4. The strains are said to be nonbiofilm producers if OD ≤ ODC [22].

### 2.7. Microbial Biofilm Inhibition Concentration

The antibiofilm potential of the antiseptics, CHG, cetrimide, PCMX, and PVP‐I on the *E. coli* and *A. baumannii* parental and adapted strains were studied using a method previously described, with modification [23, 24]. Briefly, the antiseptics are serially diluted in 2‐fold increments in 96‐microwell flat bottom plates (Cat No. 3596, Corning). The bacterial inoculum with a bacterial load of ~10^5^ CFU/mL is added to all the wells except in H‐1 to H‐12, which is used as the media control. The plates are incubated for 48 h at 37°C. Following incubation, the absorbance at 600 nm was measured using the Tecan Infinite M Plex multimode reader (Bioscreen, Tecan Trading AG, Seestrasse, Männedorf, Switzerland) to determine the growth optical density, after which the wells were washed thrice with 1x PBS for removal of the planktonic cells. The wells were then stained with 0.2% crystal violet and incubated for 15 min. The excess stain was removed by washing the wells thrice with dH_2_O, and the stained dye was resolubilized with 30% acetic acid. The solubilized dye was quantified by measuring the absorbance at 590 and 620 nm using the Tecan Infinite M Plex multimode reader (Bioscreen, Tecan Trading AG, Seestrasse, Männedorf, Switzerland). The specific biofilm formation and adhesion index was calculated by using the following formula:

Specific biofilm formation SBF=A590 biofilm−A590 blankA600,

where A_590_ (biofilm) is the absorbance at 590 nm for the test wells, A_590_ (blank) is the absorbance for the media control wells, and A_600_ is the absorbance measured at 600 nm for the planktonic cells.

Adhesion index AI=A620 testA620 control,

where A_620_ (test) is the absorbance measure at 620 nm of the resolubilized crystal violet stain used for the biofilms with antimicrobials, and A_620_ (control) is the absorbance measure at 620 nm of the resolubilized crystal violet stain used for the biofilms without antimicrobials. By measuring the absorbance at 620 nm for the test and control, a quantitative comparison of biofilm can be done. A higher A_620_ value indicates a higher stain retention translating to a stronger adhesion rate, whereas a low A_620_ value indicates a low stain retention indicating a weak adhesion rate [25].

### 2.8. Evaluation of Biofilm Formation Inhibition Using MTT

The 3‐[4,5‐dimethyl‐2‐thiazolyl]‐2,5‐diphenyl‐2H‐tetrazolium bromide (MTT) assay was performed with modifications to assess the viable bacteria within the biofilm, according to the previously described method by Kang et al. [26, 27]. Briefly, *E. coli* and *A. baumannii* parental and CHG, cetrimide, PCMX, and PVP‐I adapted strains were cultured in 96‐well flat‐bottomed microtiter plate (Cat No. 3596, Corning) with TSB plus serially diluted CHG, cetrimide, PCMX, and PVP‐I, respectively, same as in Section 2.7. The plates were incubated at 37°C for 48 h. After incubation, the planktonic cells were removed and the wells were washed gently with PBS three times to remove nonadherent bacteria. Next, 50 *μ*L of MTT solution (2 mg/mL in PBS) was added to each well, and the plates were incubated at 37°C for 3 h in the dark. Post incubation, the MTT solution was removed, and 100 *μ*L of 5% SDS buffered dimethyl sulfoxide (DMSO) was used to solubilize the formazan product. The absorbance of the dissolved formazan solution was measured at 570 nm using the Tecan Infinite M Plex multimode reader (Bioscreen, Tecan Trading AG, Seestrasse, Männedorf, Switzerland).

### 2.9. Statistical Analysis

The data in the study are presented as the mean ± standard deviation for *n* = 3 replicates. Excel was used for all the statistical analysis. The comparison between parental and adapted strain for specific biofilm formation, adhesion index, and biofilm viability for *E. coli* and *A. baumannii* were two‐tailed, and a *p* value of < 0.05 was considered statistically significant. Excel was used for plotting all the graphs in the study.

## 3. Results

### 3.1. Comparison of Antiseptic Susceptibility Pattern Among the Strains

The antimicrobial activity of CHG, cetrimide, PCMX, and PVP‐I on *E. coli* and *A. baumannii* were estimated by studying the minimum inhibitory concentrations. Among the tested antiseptics, CHG showed the lowest MIC at 0.0675 *μ*g/mL (Table 1) for *E. coli,* similar to earlier studies ([13]). Cetrimide showed the lowest MIC, 3.6 *μ*g/mL against *A. baumannii* (Table 1), which is lower than that reported for clinical strains of *A. baumannii* [28, 29]. Overall, for these two bacterial strains, the actives CHG and cetrimide had a higher antibacterial activity i.e. were effective at much lower concentrations in comparison with PCMX and PVP‐I and thus, suggests a potent antiseptic activity against *E. coli* and *A. baumannii*, over PCMX and PVP‐I in the current study.

**Table 1:** The concentrations at which *E. coli* ATCC 10536 and *A. baumannii* ATCC 19606 adapted to the tested antiseptics. The minimum inhibitory concentrations of the antiseptics against the parental strains of *E. coli* ATCC 10536 and *A. baumannii* ATCC 19606 are also presented.

Antiseptics/actives	Typical clinical concentration	Adaptation concentrations		MIC value before adaptation	
		*E. coli*	*A. baumannii*	*E. coli*	*A. baumannii*
CHG (*μ*g/mL)	3000	5	40	0.0675	7.35
CET (*μ*g/mL)	30000	22.5	27.5	0.45	3.6
PCMX (*μ*g/mL)	48000	160	400	125	125
PVP‐I (*μ*g/mL)	100000	5000	5000	1560	780

### 3.2. Adaptation of Bacterial Strains to Antiseptics

Adaptation of *E. coli* and *A. baumannii* to CHG, cetrimide, PCMX, and PVP‐I was carried out by exposure to continuous increments in the concentration of the respective antiseptics until no growth was observed. The adaptation was achieved for both the bacterial species against all the antiseptics over several passages (Table S1). Both *E. coli* and *A. baumannii* showed different adaptability profiles for each of the actives. The concentrations of antiseptics at which each bacterial strain adapted are given in Table 1. Study by Barakat et al. [30], showed that the PVP‐I adapted strains revert to their original MIC post adaptation. However, in our study CHG, cetrimide, PCMX, and PVP‐I adapted strains of *E. coli* and *A. baumannii* had the same adapted MIC values after stabilization in antiseptic‐free media postadaptation, indicating the stability of adapted strains.

### 3.3. Change in Minimum Inhibitory Concentration of the Adapted Strains

To ascertain if adaptation to a higher concentration of antiseptic translates to a higher MIC, the MIC for the adapted strains against the respective antiseptics was tested. In the current study, an adapted bacterial species is considered to be tolerant to the antiseptic if the MIC value increased by a factor of 4‐fold or more. Compared with the non‐adapted *E. coli* strain, *E. coli* adapted to cetrimide showed a significant increase in MIC value, 16‐fold (Table 2) indicating tolerance. It was observed that *E. coli* adapted to CHG exhibited a weak adaptive response with a ~1.8‐fold (Table 2) increase in MIC value. *An exception to this was E. coli adapted to* PCMX and PVP‐I with no change in MIC value from the parental strains (Table 2). A similar observation was made by Barreto et al. [31], where bacteria exhibited no significant alteration in the MIC value between the parent strain and a strain exposed to subinhibitory concentrations of povidone‐iodine ([32]).

**Table 2:** The minimum inhibitory concentrations of the studied antiseptics against *E. coli* ATCC 10536 and *A. baumannii* ATCC 19606 adapted to the antiseptics, CHG, cetrimide, PCMX, and PVP‐I. The fold increase or decrease in the minimum inhibitory concentration values for the adapted *E. coli* ATCC 10536 and *A. baumannii* ATCC 19606 with respect to the minimum inhibitory concentration values of the unadapted *E. coli* ATCC 10536 and *A. baumannii* ATCC 19606 are also represented.

Actives	MIC before adaptation	MIC value after adaptation for *E. coli*	Fold increase in the MIC
	*E. coli*		
CHG (*μ*g/mL)	0.0675	0.125	1.8
Cetrimide (*μ*g/mL)	0.45	7.2	16
PCMX (*μ*g/mL)	125	125	No change
PVPI (*μ*g/mL)	1560	1560	No change
	***A. baumannii***	**MIC value after adaptation of *A. baumannii***	**Fold increase in the MIC**
CHG (*μ*g/mL)	7.35	14.7	2
Cetrimide (*μ*g/mL)	3.6	3.6	No change
PCMX (*μ*g/mL)	125	125	No change
PVPI (*μ*g/mL)	780	3130	4

The PVP‐I adapted strain of *A. baumannii* can be defined as PVP‐I tolerant strain, as its MIC value increased by 4‐fold (Table 2). *A. baumannii* adapted to cetrimide and PCMX exhibited no change in their MIC values in comparison to the parental strain. The results indicate that adaptation in bacteria towards antiseptics is differential and variable in nature and changes between bacterial species studied. It should be noted that despite showing a higher MIC, the adapted strains remain susceptible to the clinically used concentrations of the actives, which are much higher than the concentrations used in the current study.

### 3.4. Cross‐Tolerance of Adapted Strains to Antiseptics

To determine whether adaptation to CHG, cetrimide, PCMX, or PVP‐I, leads to development of cross‐tolerance or susceptibility to the other studied antiseptics, the MIC was determined for all the adapted strains for the corresponding non‐adapted antiseptics (Table 3). In the study, an adapted bacterial species is considered to be cross‐tolerant to another antiseptic if the MIC value increased by a factor of 4‐fold or more.

**Table 3:** The minimum inhibitory concentrations for the studied antiseptics against the bacterial strains of *E. coli* ATCC 10536 and *A. baumanii* ATCC 19606 adapted to CHG, cetrimide, PCMX, and PVPI, each using crystal violet assay.

Actives	Control	CHG‐adapted strain	Cetrimide‐adapted strain	PCMX‐adapted strain	PVPI‐adapted strain
*E. coli*					
CHG (*μ*g/mL)	0.0675	**0.125**	0.5625	0.465	0.465
Cetrimide (*μ*g/mL)	0.45	3.6	**7.2**	0.93	3.6
PCMX (*μ*g/mL)	125	125	125	**125**	125
PVPI (*μ*g/mL)	1560	1560	1560	1560	**1560**
*A. baumannii*					
CHG (*μ*g/mL)	7.35	**14.7**	7.35	3.6	7.35
Cetrimide (*μ*g/mL)	3.6	3.6	**3.6**	14.7	3.6
PCMX (*μ*g/mL)	125	62.5	93.75	**125**	125
PVPI (*μ*g/mL)	780	780	780	6	**3130**

*Note:* Values in bold indicate the MIC values of the antiseptics against the same antiseptic adapted *E. coli* and *A. baumannii*, respectively.

In the case of *E. coli,* strains adapted to CHG and cetrimide showed an approximately 10‐fold decrease in susceptibility towards cetrimide and CHG (Table 3), respectively, while maintaining its susceptibility towards PCMX and PVP‐I same as the parental strain (Table 3). PCMX and PVP‐I adapted *E. coli* strains, showed a higher MIC for CHG, a 6‐fold increase (Table 3) indicating cross‐tolerance towards CHG. In the case of *A. baumannii*, CHG, cetrimide, and PVP‐I adapted *A. baumannii* showed no change in susceptibility towards the tested antiseptics. Notably, only *A. baumannii* adapted to PCMX demonstrated an increased susceptibility (~130‐fold) towards PVP‐I (Table 3). This data indicates that *E. coli* adapted strains generated an increased cross‐tolerance to the cationic compounds CHG and cetrimide in comparison to the *A. baumannii* adapted strains.

### 3.5. Antibiotic Cross‐Resistance

**Figure 2:**
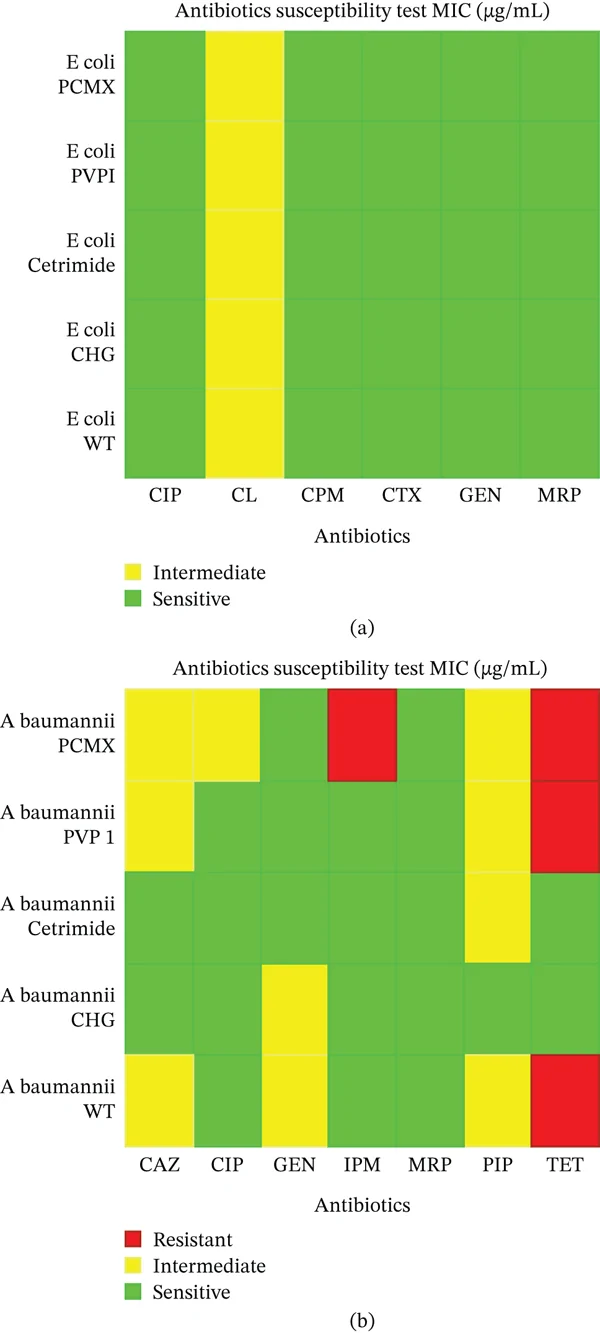
Antibiogram of (a) *E. coli* ATCC 10536 and (b) *A. baumannii* ATCC 19606 parental strains and antiseptic adapted strains. Antibiotics used: GEN, gentamicin; CIP, ciprofloxacin; TET, tetracycline; IPM, imipenem; PIP, piperacillin; CAZ, ceftazidime; MRP, meropenem; CTX, cefotaxime; CL, colistin; and CPM, cefepime.

The adapted strains of *E. coli* showed no cross‐resistance to any of the antibiotics tested. The MIC values for the tested antibiotics were either similar or insignificantly changed (≤ 2‐fold increase/decrease in MIC) compared with the parental strain (Table S2). The antibiotic susceptibility pattern for all the adapted *E. coli* strains were similar to that of the parental *E. coli* (Figure 2a).

*A. baumannii* adapted strains, which did not demonstrate a strong deviation from the parental MICs for antiseptic cross tolerance, showed a significant change in the antibiotic MIC values compared with the parental strain. The PCMX adapted *A. baumannii* strain demonstrated a significantly higher MIC value for tetracycline (> 256 *μ*g/mL) and imipenem (> 32 *μ*g/mL) (Table S3). Thus, adaptation of *A. baumannii* to PCMX has led to a cross‐resistance towards Imipenem, which belongs to the Carbapenem class of antibiotics which is usually used as the first line of antibiotics against *A. baumannii* infections [33]. Surprisingly, CHG adapted *A. baumannii* demonstrated a significant decrease in the MIC values for all the antibiotics, which resulted in the strain becoming sensitive to ceftazidime, piperacillin, and tetracycline (Table S3). The cetrimide‐adapted *A. baumannii* also showed an increased susceptibility with lower MIC values for all the tested antibiotics and exhibited sensitivity for ceftazidime, tetracycline, and gentamicin (Figure 2b and Table S3).

### 3.6. Impact of Antiseptic Adaptation on *E. coli* and *A. baumannii* Biofilm Formation

Biofilm formation is one of the major strategies that aids in the development of resistance in various nosocomial pathogens. *A. baumannii* and *E. coli* both have the capacity to develop robust biofilms on abiotic as well as on biotic surfaces. The biofilms help *E. coli* and *A. baumannii* to resist the action of antimicrobial agents including antiseptics [34, 35]. Owing to the role played by the bacterial biofilm in driving antimicrobial resistance, the biofilm forming ability of the adapted strains was assessed using quantitative biofilm assay. *E. coli* and its antiseptic adapted bacterial strains were inferred to be strong biofilm producers, as the OD_590nm_ was > 4 times the ODC value [22] (Table S4). The same trend was observed for *A. baumannii* and its adapted strains (Table S4).

Next, we determined the specific biofilm formation value for each strain. We observed that the specific biofilm formation in the adapted strains of *E. coli* CHG, *E. coli* cetrimide, *E. coli* PCMX, and *E. coli* PVP‐I remained the same, (≤ 2‐fold) (Figure S1) in comparison to the parental strain. A similar result was observed for the CHG, cetrimide PCMX, and PVP‐I adapted strains of *A. baumannii*, i.e. no change (≤ 2‐fold) (Figure S1) in the biofilm formation compared with the parental strain was observed.

### 3.7. Inhibition of Biofilm Formation by Antiseptics in *E. coli* and *A. baumannii* Adapted Strain

**Figure 3:**
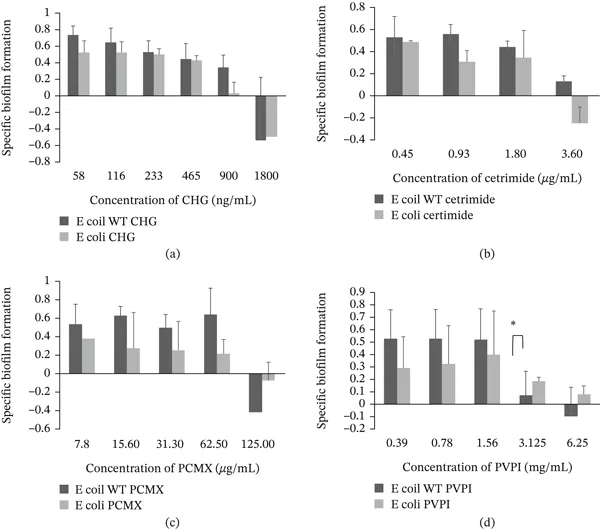
Comparison of the specific biofilm formation capability between *E. coli* ATCC 10536 parental strain and the antiseptic adapted strains when treated with (a) CHG, (b) cetrimide, (c) PCMX, and (d) PVP‐I. Statistically significant difference between parental and adapted bacterial strains is marked with “∗” (0.1 ≤ *p* ≥ 0.05).

Next, ability of the antiseptics to inhibit the biofilm formation in the adapted strains was evaluated. The adapted *E. coli* CHG, cetrimide, and PCMX strains retained the biofilm forming capacity comparable to the parental strains in the presence of the respective actives (Figure 3a–c). In contrast, biofilm formation in PVP‐I adapted *E. coli* was inhibited at a higher concentration of PVP‐I in comparison to the parental strain (*p* = 0.057). Adhesion index quantifies the capacity of bacterial cells to adhere to substrates/surfaces and form biomass relative to their overall planktonic growth. In the assay, as crystal violet nonspecifically binds to bacterial cell walls and extracellular polymeric substance (EPS) matrix, calculating the adhesion index normalizes the absorbance against cell density, depicting a true anti‐adherence activity. A dose‐dependent decrease in the adhesion index value demonstrates that the antiseptic agent targets the initial biofilm formation interactions, thereby arresting the developmental cascade of bacterial biofilm prior to stable matrix formation. Cetrimide‐adapted *E. coli* strain showed a significant increase in the adhesion index value in comparison to the treated parental strain (Figure S2b), that is, the cetrimide‐adapted *E. coli* strain has a higher tendency to attach to the substrate. MTT assay, used to assess the metabolic activity of bacteria cells within the biofilms, was used to evaluate the viability of the biofilms formed by the parental and adapted strains of *E. coli*. The CHG and PCMX adapted *E. coli* showed no change in biofilm viability from the parental strain (Figure S3a,c). The cetrimide and PVP‐I adapted *E. coli* showed statistically significant increase in biofilm viability when compared with the parental strain (Figure S3b,d). The adapted *E. coli* strains showed similar biofilm inhibition concentrations as that of the parental strains when treated with same actives. And, all the adapted *E. coli* strains got inhibited well below the clinical concentrations.

**Figure 4:**
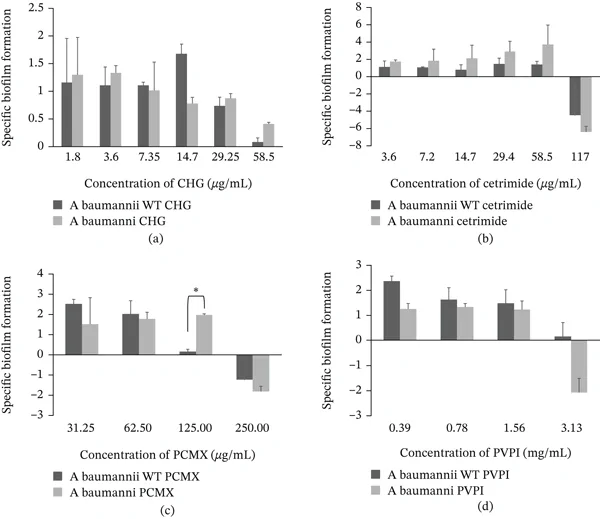
Comparison of the specific biofilm formation capability between *A. baumannii* ATCC 19606 parental strain and the antiseptic adapted strains when treated with (a) CHG, (b) cetrimide, (c) PCMX, and (d) PVP‐I. Statistically significant differences between parental and adapted bacterial strains are marked with “∗” (0.05 ≤ *p* ≥ 0.01).

In case of *A. baumannii*, there was no statistically significant change (*p* > 0.05) observed in the reduction of biofilm by CHG, cetrimide, and PVP‐I between the parental and the respective adapted *A. baumannii* strains (Figure 4a,b,d). *A. baumannii* PCMX exhibited a significant (*p* = 0.028) increase in the biofilm formation in comparison with the parental strain (Figure 4c). Unlike specific biofilm formation, the adhesion index value for all the adapted strains of *A. baumannii* exhibited no change in comparison to the parental *A. baumannii* strain (Figure S4a,b,c,d).

The biofilm viability for cetrimide and PVP‐I adapted *A. baumannii* was similar to that of the parental strain (Figure S5b,d). In contrast, the CHG and PCMX adapted *A. baumannii* showed a statistically significant increase in biofilm viability in comparison to the parental *A. baumannii* (Figure S5a,c). Except for the *A. baumannii* PCMX adapted strain, all the adapted *A. baumannii* strains had final biofilm inhibition concentrations similar to that of the parental strain. Nevertheless, all the adapted *A. baumannii* strains were inhibited below the clinical concentrations.

## 4. Discussion

Antiseptics usage is seeing rapid growth, causing concerns of bacterial tolerance towards the antiseptics and cross‐resistance towards other antimicrobials. Although literature reports show that in vitro adapted bacteria display decreased susceptibilities towards antiseptics, in reality, these lab‐adapted bacteria remain effectively susceptible towards the clinical concentration of the antiseptics. Previous studies indicate that continuous exposure of bacteria to antiseptics may not only lead to selection of antiseptic‐resistant mutants with stable phenotypes but may also give rise to reversible phenotypic adaptations as consequence of temporary change in gene expression due to stress response [36]. This adds a layer of complexity to the diversity within the mechanisms of action of antiseptics. Thus, when a bacterium is exposed to target site‐specific antiseptics like triclosan, it can lead to selection of mutations at the target proteins. However, induced insusceptibility towards membrane‐active compounds such as biguanides and quaternary ammonium compounds is often associated with induction of stress responses [36, 37]. In this in vitro study, *E. coli* and *A. baumannii*, two of the major organisms causing nosocomial infections in the current healthcare systems [38, 39], were studied to analyze their potential to gain tolerance towards four distinct antiseptics—chlorhexidine, cetrimide, chloroxylenol, and povidone iodine.

### 4.1. Antiseptic Exposure Induces Changes in Antimicrobial Susceptibility and Cross‐Susceptibility

The results in this study highlight that exposure to antiseptics influences antiseptic susceptibility in *E. coli* and *A. baumannii* and this change depends on the species of bacteria. Chlorhexidine, a biguanide is known to target bacterial cytoplasmic membrane. Change in the bacterial susceptibility pattern towards CHG is usually achieved through change in expression of multidrug resistance efflux pumps and alterations in the structural integrity of bacterial cell envelope, which impacts permeability of the cells [40–42]. In the current study, the parental strains of *E. coli* and *A. baumannii* both exhibited susceptibility towards CHG well within the limit of clinically used concentrations. Although they adapted at concentrations much higher than their initial MICs, both the adapted strains showed only a moderate 1.8–2‐fold increase in MIC value in comparison to the parental strain. Thus, even though reports suggest that widespread use of chlorhexidine is associated with increasing cases of reduced susceptibility in various nosocomial infection causing bacteria, including *K. pneumoniae*, *Pseudomonas aeruginosa*, and *A. baumannii* [43], we did not observe significant changes in the current study. It was also observed that each of the CHG‐tolerant strains of *E. coli* and *A. baumannii* showed unique cross‐susceptibility patterns for the studied antiseptics. For example, *E. coli* CHG strain became more tolerant to cetrimide, whereas *A. baumannii* CHG strain showed a 2‐fold increased susceptibility to PCMX, emphasizing the role played by respective bacterial species, their structural composition, and changes in genes involved with various efflux pumps and metabolic pathways [44] in developing cross‐tolerance to antiseptics. The study by Daller et al. [45] on understanding the genomic and transcriptomic mechanisms of CHG adaptation in *Streptococcus* spp. revealed mutations in genes related to membrane structure, DNA repair, and metabolic processes. For example, *Streptococcus mitis* adapted to CHG showed a mutation in polyamine transporter *potA*, which is part of the potABCD system that plays a pivotal role in the oxidative stress response. Bacterial tolerance to disinfectants is also thought to be related to chromosomal mutations. A study by Pereira et al. [46] showed that overexpression of the *yahD* gene in *E. coli* leads to an increased tolerance towards glutaraldehyde. Cetrimide is a cationic surfactant, which acts by disrupting microbial cell membranes. In this adaptation study, we observed *E. coli* cetrimide strain developed a significant increase in cross‐tolerance towards CHG. This could be either attributed to the structural changes or due to an increased efflux activity, helping bacteria expel antiseptic from the cell faster [1, 40]. Further studies with a combination of cetrimide and CHG are of high clinical relevance since these antiseptics are often used in combination. Chloroxylenol (PCMX), a widely used phenolic antiseptic is known to disrupt the bacterial cell wall and is most potent against gram‐positive bacteria. In our study, these gram‐negative bacteria adapted to PCMX at higher concentrations with no alteration in MIC. PCMX adapted *E. coli* showed a cross‐tolerance to CHG with a ~6‐fold increase in MIC than the parental strain. Interestingly, the PCMX adapted *A. baumannii* showed a significantly higher sensitivity to PVP‐I (130‐fold decrease in MIC value). Although there are studies highlighting decrease in susceptibility towards a particular antiseptic after long‐term exposures [13, 17, 36], fewer studies report increased cross‐susceptibility due to antiseptic exposure. Further research is needed to understand the underlying mechanism for such heightened cross‐susceptibility or sensitivity towards another antiseptic. Unlike, CHG, cetrimide, and PCMX, PVP‐I has a well‐established antimicrobial activity against gram‐positive, gram‐negative, and some spore forming bacteria and mycobacteria [47]. PVP‐I tolerance in bacteria is seldom reported and the same is evident in the current study as well. However, like in the instances above, PVP‐I adapted *E. coli* shows cross‐adaptation to other antiseptics—for example, nearly10‐fold increase in MIC of cetrimide and CHG as compared with the parental strains. Change in susceptibility of bacterial species to antiseptics is marked by structural variations in the bacterial cell. These variations (a) impact the binding of antiseptics to the cell, (b) alter cell permeability, and (c) cause modification in efflux pumps [48, 49]. The changes in antiseptic susceptibility in the study may also be attributed to these reasons; however, the exact mechanism that governs each specific tolerance will likely depend on factors inherent to the bacteria and the antiseptic combination, and needs to be explored further.

### 4.2. Adaptation of *A. baumannii* Induces Bidirectional Shift in Antibiotic Susceptibility

There is growing evidence of cross‐resistance between antiseptics and antibiotics. Mutations acquired from the use of antiseptics or antibiotics can lead to cross‐resistance to either. These mutations may be encoded on the same genetic elements such as transposons, plasmids, or integrons. Previous studies in staphylococci showed that mutations in the genes lying on plasmids *qacAB* and *qacCD* cause resistance against QACs and against antibiotics [44]. For instance, in *S. aureus* the qacA/B genes occur frequently on the pSK1 and *β*‐lactamase/heavy metal‐resistance plasmids that are also known to confer resistance to antibiotics [50, 51]. Similarly, Weigel et al. [52] reported a linkage between trimethoprim (dfrA), aminoglycosides (aacA‐aphD), *β*‐lactams (blaZ), and antiseptics (qacC) mediated by a multiresistance plasmid in staphylococci. Similar to staphylococci, Qac genes in Enterobacteriaceae are also found in combination with genes that code for resistance to sulphonamides, aminoglycosides, *β*‐lactams, chloramphenicol, and trimethoprim [50].

In the current study, we observed acquisition of both antibiotic resistance and sensitivity in the antiseptic adapted strains of *A. baumannii*. Improved susceptibility or antibiotic sensitivity was observed for *A. baumannii* CHG, cetrimide, and PVP‐I strains. *A. baumannii* CHG strain became susceptible to tetracycline, ceftazidime, and piperacillin; *A. baumannii* cetrimide strain became susceptible to tetracycline, ceftazidime, and gentamicin; whereas *A. baumannii* PVP‐I strain showed susceptibility towards gentamicin. The potential of antiseptics CHG and cetrimide to initiate the fitness trade‐off towards a positive outcome, that is, a higher antibiotic sensitivity is an attractive and promising attribute when considering their use in hospitals. On the other hand, we observed that PCMX adaptation on one hand induced cross‐resistance towards imipenem in the previously susceptible *A. baumannii* strain, whereas it increased susceptibility towards gentamicin. Previous studies on imipenem resistance in clinical isolates of *A. baumannii* reveals that resistance in *A. baumannii* MDR strains are regulated by a system of efflux pumps including AdeABC, AdeIJK, and AbeM ([53]). Among these AdeABC and AdeIJK, belonging to the resistance‐nodulation‐cell division (RND) superfamily are known to mediate intrinsic and acquired tolerance to antiseptics [54] and might be involved with the cross‐resistance seen in *A. baumannii*. Interestingly, *A. baumannii* PCMX strain also showed a significantly higher MIC for tetracycline, ~10‐fold higher than the parental strain. Tetracycline resistance in *A. baumannii* is coregulated by the genes *tetA, tetB,* and *adeABC* [55], of which *adeABC* binds to a wide range of substrates including fluoroquinolones, macrolide, *β*‐ lactams, and chloramphenicol [56]. Thus, an increased MIC for tetracycline through AdeABC efflux system may indicate alteration in the antimicrobial resistance profile for other antimicrobials. Though the genetic elements for antibiotic resistance in clinical isolates of *A. baumannii* are known, the role played by these genes in inducing resistance in alternative antimicrobials, and their role in bacterial fitness trade‐off remains unexplored and requires further exploration.

### 4.3. Alteration in Biofilm Formation Due to PCMX Adaptation

*A. baumannii* is known for its ability to produce biofilm, which helps it to survive extremely harsh environments and resist antimicrobials [57]. Studies show that *A. baumannii* adapted to antiseptics exhibit phenotypic changes including altered biofilm formation, growth rate, and competitive fitness and that these factors define the pathogenicity and virulence potential of the adapted bacteria [41, 58]. Among the *A. baumannii* adapted strains in this study, only *A. baumannii* PCMX strain exhibited a significant change in biofilm formation post treatment. The MTT assay established the improved biofilm stability and viability of the strain *A. baumannii* PCMX under higher concentration of PCMX in comparison to the parental strain of *A. baumannii*. This change corresponds with its increased resistance to imipenem antibiotic. Previous literature states that *Acinetobacter* spp. known to produce biofilm harbor at least one of the following virulence genes—*ompA, bap, bfmS*, and *epsA* [59]. The *bap* gene expressed on bacterial cell surface is responsible for biofilm formation on abiotic/biotic surfaces [60]. Further investigation on genetic mutations and expression level changes of these genes, especially *bap* genes is required to understand the mechanism for formation of a stronger biofilm in *A. baumannii* PCMX.

### 4.4. Low Level Adaptation and Cross Adaptation of Bacteria to PVP‐I

Povidone iodine is a widely used antiseptic in clinical settings, including for skin disinfection pre‐ and post surgery. PVP‐I is known to have a broad antimicrobial spectrum and is active against antibiotic and antiseptic‐resistant bacteria [61]. Safety and efficacy data for PVP‐I supports its suitability in usage against bacterial biofilms [62]. In the current study, *E. coli* and *A. baumannii*, both adapted to PVP‐I at concentrations higher than initial MICs but only *A. baumannii* showed a 4‐fold increase in the MIC post adaptation, making it a PVP‐I tolerant strain. This corroborates with the existing literature, where development of tolerance against PVP‐I has not been observed. This might be due to its numerous and simultaneous molecular targets—amino groups, double bonds, and sulfhydral groups [61]. Unlike previously reported studies, the current adaptation study yielded stable adapted strains of *E. coli* and *A. baumannii* against PVP‐I, facilitating antiseptic and antibiotic cross‐resistance studies. Here, we observed cross‐tolerance in *E. coli* PVP‐I adapted strain towards CHG (~6‐fold increase in MIC) and cetrimide (~8‐fold increase). We also evaluated the association between PVP‐I adaptation and antibiotic resistance profile in both *E. coli* and *A. baumannii*. The study revealed that *A. baumannii* became sensitive to gentamicin on adaptation to PVP‐I. Our study reiterates the findings that PVP‐I, being a broad‐spectrum antiseptic targeting multiple sites, does not induce cross‐resistance towards antiseptic and antibiotics on long‐term continuous exposure.

## 5. Conclusion

In conclusion, our study ascertains that continuous in vitro exposure of bacteria to antiseptics can induce antibiotic cross‐resistance and altered biofilm dynamics. The clinical implications of antiseptic induced antibiotic cross‐resistance can be substantial, particularly in healthcare settings where antiseptics are used routinely and extensively. We demonstrate that sublethal exposure to antiseptics can act as a selective pressure promoting cross‐adaptation to both antiseptics and antibiotics, although the resulting outcomes are dependent on the specific combination of bacterial species, antiseptic, and antibiotic. It has been reported that changes in susceptibility seen in adapted bacteria might be either sustained or transient depending on selection of stable genetic mutations or temporary phenotypic adaptation, respectively [41], and these vary between bacterial species. In our study, frontline antiseptics like CHG and PVP‐I continued to demonstrate their efficacy in vitro, despite adaptation in the *E. coli* and *A. baumannii* strains. Nevertheless, future studies employing multiomics approaches combining whole genome sequencing and RNA sequencing are warranted to delineate the molecular basis of cross‐resistance, particularly those associated with efflux pumps and membrane modifications that could contribute to this phenotype.

Our study provides an evidence‐based framework for disinfectant usage by providing concentration ranges that preserve bactericidal efficacy while eliminating suboptimal concentrations that exert selective pressure that eventually promote bacterial adaptation and cross‐resistance. Along with the molecular and analytical studies investigating antiseptic tolerance, further research is needed to examine its emergence and propagation in real‐world clinical settings. This gap between laboratory observations and clinical infection control can be addressed through multicenter longitudinal surveillance studies that monitor antiseptic susceptibility trends among hospital surface and clinical isolates over time.

Future research on antiseptic led antibiotic resistance in clinical settings should also investigate modified infection control practices that prevent or reverse the emergence of antiseptic tolerance. Controlled hospital‐based field studies that evaluate whether alternating antiseptic regimens or application of antiseptic combinations effectively prevent resistance emergence and help in sustaining the utility of critical antibiotic therapies also need to be seen.

Tackling antiseptic resistance and monitoring its impact on antimicrobial resistance, are critical for advancing the One Health approach, which seeks to sustainably safeguard the health of humans, animals, and ecosystem Thus, surveillance of resistance across these interconnected sectors will advance our understanding of AMR evolution and dissemination and will facilitate the development of effective prevention and control strategies.

**Nomenclature**
CHG: chlorhexidine gluconate
PCMX: chloroxylenol
PVP‐I: povidone iodine
QACs: quaternary ammonium compounds

## Funding

No external funding was provided or received for this manuscript.

## Conflicts of Interest

All the authors of the present study are affiliated to the ITC Life Sciences and Technology Centre, Bengaluru, India, as salaried employees. The ITC Sciences and Technology Centre is under the aegis of ITC Limited.

## Supporting information


**Supporting Information** Additional supporting information can be found online in the Supporting Information section. Table S1: The number of passages it took for *E. coli* and *A. baumannii* to adapt to chlorhexidine, cetrimide, chloroxylenol, and povidone iodine, respectively. Table S2: Summary of MIC values for each adapted strain of *E. coli* against the antibiotics. Table S3: Summary of MIC values for each adapted strain of *A. baumannii* against the antibiotics. Table S4: Classification of parental and adapted *E. coli* and *A. baumannii* strains to strong, moderate, and weak biofilm formers. Figure S1: Biofilm formation in parental and adapted strains of *E. coli* and *A. baumannii* in untreated condition. Figure S2: Comparison of adhesion index value between *E. coli* parental strain and the adapted strains. Figure S3: Comparison between *E. coli* parental strain and the adapted strains in forming viable biofilms. Figure S4: Comparison of adhesion index value between *A. baumannii* parental strain and the adapted strains. Figure S5: Comparison between *A. baumannii* parental strain and the adapted strains in forming viable biofilms.

## Data Availability

The datasets that support the findings in the study are available from the corresponding author upon reasonable request.
