# Supplementary material for: Impact of Incremental Antiseptic Selection on Antibiotic Cross‐Resistance and Enhanced Biofilm Formation in Escherichia coli and Acinetobacter baumannii: In Vitro
Source: Int J Microbiol. 2026 Sep 26;2026:5044102. doi: 10.1155/ijm/5044102 (PMC13615512; doi:10.1155/ijm/5044102)
Supplement: Supplementary file 1 [file IJM-2026-5044102-s001.docx]

**Supplementary File**

**Impact of incremental antiseptic selection on antibiotic cross-resistance and enhanced biofilm formation in *Escherichia coli* and *Acinetobacter baumannii, in vitro***

Megha Prasad^1^, Pratheesh N Prabhakaran^1^, Kulpreet Bhui^1*^ and Deepa Murali^1*^

| **Bacteria** | ***E coli* CHG** | ***E coli* Cetrimide** | ***E coli* PCMX** | ***E coli* PVPI** |
| --- | --- | --- | --- | --- |
| **No. of passages** | 9 | 8 | 4 | 5 |
| **Concentration progression (μg/mL)** | 0.03, 0.06, 0.12, 0.25, 0.5, 1, 2, 4, 5 | 0.225, 0.45, 1, 5, 10, 20, 22, 22.5 | 62.5, 125, 150, 160 | 780, 1560, 3120, 4000, 5000 |
| **Bacteria** | ***A baumannii* CHG** | ***A baumannii* Cetrimide** | ***A baumannii PCM*X** | ***A baumannii* PVPI** |
| **No. of passages** | 7 | 9 | 10 | 7 |
| **Concentration progression (μg/mL)** | 3.6, 7.35, 14.5, 30, 35, 35, 40 | 1.8, 3.6, 7.5, 14, 20, 22.5, 25, 26, 27.5 | 62.5, 125, 150, 175, 200, 250, 300, 350, 375, 400 | 390, 780, 1560, 3000, 4000, 4500,5000 |

**Table S1:** The table indicates the number of passages it took for *E coli* and *A baumannii* to adapt to chlorhexidine, cetrimide, chloroxylenol and povidone iodine, respectively.

| Bacteria | MIC (µg/mL) | | | | |  |
| --- | --- | --- | --- | --- | --- | --- |
|  | **GEN** | **CTX** | **MRP** | **CIP** | **CL** | **CPM** |
| *E. coli* WT | 0.5 | 0.023 | 0.023 | 0.023 | 0.75 | 0.19 |
| *E. coli* CHG | 0.38 | 0.023 | 0.032 | 0.032 | 0.75 | 0.19 |
| *E. coli* Cetrimide | 0.5 | 0.032 | 0.023 | 0.023 | 0.75 | 0.25 |
| *E. coli PVP-I* | 0.5 | 0.047 | 0.047 | 0.032 | 1.5 | 0.35 |
| *E. coli* PCMX | 0.75 | 0.064 | 0.047 | 0.047 | 1 | 0.38 |

**Table S2. Summary of MIC values for each adapted strains of *E. coli* against the antibiotics.** The antibiotics used are GEN- Gentamicin; CIP- Ciprofloxacin; MRP- Meropenem; CTX- Cefotaxime; CL- Colistin; CPM- Cefepime.

| Bacteria | MIC (µg/mL) | | | | | | |
| --- | --- | --- | --- | --- | --- | --- | --- |
|  | **GEN** | **MRP** | **CIP** | **TET** | **CAZ** | **PIP** | **IPM** |
| *A. baumannii* WT | 6 | 1.5 | 1 | 24 | 16 | 48 | 2 |
| *A. baumannii* CHG | 6 | 0.125 | 0.19 | 4 | 0.19 | 8 | 0.5 |
| *A. baumannii* Cetrimide | 2 | 0.5 | 0.75 | 4 | 0.75 | 24 | 0.75 |
| *A. baumannii* PVP-I | 3 | 0.75 | 0.5 | 24 | 24 | 48 | 1.5 |
| *A. baumannii* PCMX | 1.5 | 2 | 1.5 | >256 | 12 | 32 | >32 |

**Table S3. Summary of MIC values for each adapted strains of *A. baumannii* against the antibiotics.** The antibiotics used are GEN- Gentamicin; CIP- Ciprofloxacin; TET- Tetracycline; IPM- Imipenem; PIP- Piperacillin; CAZ- Ceftazidime, MRP- Meropenem;

| S. no. | Bacteria | OD 590nm | ODC | Fold Increase | Type of Biofilm formation |
| --- | --- | --- | --- | --- | --- |
| *E. coli* | | | | | |
| 1 | ***E. coli* WT** | 0.692 | 0.047 | 14.5 | Strong |
| 2 | ***E. coli* CHG** | 0.512 | 0.044 | 11.6 | Strong |
| 3 | ***E. coli* CETRIMIDE** | 0.635 | 0.047 | 13.5 | Strong |
| 4 | ***E. coli* PCMX** | 0.655 | 0.043 | 14.9 | Strong |
| 5 | ***E. coli* PVP-I** | 0.532 | 0.047 | 11.1 | Strong |
| *A. baumannii* | | | | | |
| 6 | ***A. baumannii* WT** | 0.702 | 0.085 | 8.21 | Strong |
| 7 | ***A. baumannii* CHG** | 0.633 | 0.135 | 4.68 | Strong |
| 8 | ***A. baumannii* CETRIMIDE** | 0.668 | 0.057 | 11.54 | Strong |
| 9 | ***A. baumannii* PCMX** | 0.748 | 0.088 | 8.42 | Strong |
| 10 | ***A. baumannii* PVP-I** | 0.67 | 0.106 | 6.3 | Strong |

**Table S4. Quantitative Biofilm formation**. The table states the values that helps categorizes the parental *E. coli* ATCC 10536 & *A. baumannii* ATCC 19606 and its adapted strains in into strong or weak biofilm formers. The strains were classified as strong biofilm producers if their OD > ODC × 4; moderate biofilm producers if 2 × ODC < OD ≤ 4 × ODC; weak biofilm producers if ODC < OD ≤ 2 × ODC, and non-biofilm producers if OD ≤ ODC. OD – Optical density and ODC – Optical density cut-off value.

**Figure S1.** **Biofilm formation in adapted strains of *E. coli* and *A. baumannii***. Specific Biofilm Formation in a. *E. coli* wildtype, *E. coli* CHG, *E. coli* Cetrimide, *E. coli* PCMX and *E. coli* PVP-I; b. *A. baumannii* wildtype, *A. baumannii* CHG, *A. baumannii* Cetrimide, *A. baumannii* PCMX and *A. baumannii* PVP-I, in untreated condition. Statistically significant difference between parental and adapted bacterial strains are marked with ‘*’ (0.05 ≤ p ≥ 0.01).

**Figure S2.** **Comparison of adhesion index value.**  The graphs compare the adhesion index value between *E. coli* parental strain and the adapted strains when treated with a) CHG, b) Cetrimide, c) PCMX, and d) PVP-I. Statistically significant difference between parental and adapted bacterial strains are marked with ‘*’ (0.05 ≤ p ≥ 0.01).

**Figure S3.** **Comparison on biofilm viability.** The graph compares the biofilm viability between *E. coli* parental strain and the adapted strains when treated with a) CHG, b) Cetrimide, c) PCMX, and d) PVP-I. Statistically significant difference between parental and adapted bacterial strains are marked with ‘*’ (0.05 ≤ p ≥ 0.01).

**Figure S4.** **Comparison on adhesion index value.** The graph compares the adhesion index between *A. baumannii* parental strain and the adapted strains when treated with a) CHG, b) Cetrimide, c) PCMX, and d) PVP-I.

**Figure S5.** **Comparison on biofilm viability.** The graph compares the biofilm viability between *A. baumannii* parental strain and the adapted strains when treated with a) CHG, b) Cetrimide, c) PCMX, and d) PVP-I. Statistically significant difference between parental and adapted bacterial strains are marked with ‘*’ (0.05 ≤ p ≥ 0.01).
